# Content counts, but context makes the difference in developing expertise: a qualitative study of how residents learn end of shift handoffs

**DOI:** 10.1186/s12909-018-1350-8

**Published:** 2018-11-03

**Authors:** Nicholas A. Rattray, Patricia Ebright, Mindy E. Flanagan, Laura G. Militello, Paul Barach, Zamal Franks, Shakaib U. Rehman, Howard S. Gordon, Richard M. Frankel

**Affiliations:** 1 VA HSR&D Center for Health Information and Communication, Roudebush VAMC, Indianapolis, USA; 2 Department of Anthropology, Indiana University-Purdue University Indianapolis, Indianapolis, USA; 3 Regenstrief Institute, Inc., Indianapolis, USA; 4 Indiana University School of Nursing, Indianapolis, USA; 5 Applied Decision Science, LLC, Dayton, USA; 6 Wayne State University School of Medicine, Detroit, USA; 7 Phoenix VA Healthcare Systems, Phoenix, USA; 8 University of Arizona College of Medicine-Phoenix, Phoenix, USA; 9 VA HSR&D Center of Innovation for Complex Chronic Healthcare, Jesse Brown VAMC, Chicago, USA; 10 University of Illinois at Chicago, Chicago, USA; 11 Indiana University School of Medicine, Indianapolis, USA

**Keywords:** Communication, Resident handoffs, Qualitative research, Resident training, Continuing education, Quality of care, Patient safety

## Abstract

**Background:**

Handoff education is both formal and informal and varies widely across medical school and residency training programs. Despite many efforts to improve clinical handoffs, little evidence has shown meaningful improvement. The objective of this study was to identify residents’ perspectives and develop a deeper understanding on the necessary training to conduct safe and effective patient handoffs.

**Methods:**

A qualitative study focused on the analysis of cognitive task interviews targeting end-of-shift handoff experiences with 35 residents from three geographically dispersed VA facilities. The interview data were analyzed using an iterative, consensus-based team approach. Researchers discussed and agreed on code definitions and corresponding case examples. Grounded theory was used to analyze the transcripts.

**Results:**

Although some residents report receiving formal training in conducting handoffs (e.g., medical school coursework, resident boot camp/workshops, and handoff debriefing), many residents reported that they were only partially prepared for enacting them as interns. Experiential, practice-based learning (i.e., giving handoffs, covering night shift to match common issues to handoff content) was identified as the most suited and beneficial for delivering effective handoff training. Six skills were described as critical to learning effective handoffs: identifying pertinent information, providing anticipatory guidance, applying acquired clinical knowledge, being concise, incorporating delivery strategies, and appreciating the styles/preferences of handoff recipients.

**Conclusions:**

Residents identified the immersive performance and the experience of covering night shifts as the most important aspects of learning to execute effective handoffs. Formal education alone can miss the critical role of real-time sense-making throughout the process of handing off from one trainee to another. Interventions targeting senior resident mentoring and night shift could positively influence the cognitive and performance capacity for safe, effective handoffs.

**Electronic supplementary material:**

The online version of this article (10.1186/s12909-018-1350-8) contains supplementary material, which is available to authorized users.

## Background

End-of-shift patient handoffs, also known as transfers-of-care or sign-outs, pose a substantial patient safety risk and an opportunity for quality improvement. These transitions lead to unwanted variation in handoffs and have been associated with delays in diagnosis and treatments [1], duplication of tests or treatment and patient discomfort [2], inappropriate care and less functional training for health care personnel [3], medication errors and failure to follow a patient’s code status [4], and longer hospital stays and increased laboratory testing [5, 6]. The Accreditation Council for Graduate Medical Education (ACGME) requires teaching hospitals to develop residents’ competency in communicating with team members during the handoff process [7]. Proposed curricula for medical students and residents in end-of-shift handoffs [8], as well as interventions that improve transfer processes, have resulted in meager changes in practice and growing awareness that one size of training does not fit all kinds of handoffs [9, 10]. Training for end-of-shift handoff competency is infrequently included in formal medical education and, where it is, the content and structure of the training varies widely [11]. Instead, end-of-shift handoffs are typically learned “on the job,” from interns or residents, who likely learned them in the same manner [12]. The clash between formal training and local practice culture (i.e., “the way things are done around here”) may contribute to the considerable variation observed in handoff effectiveness [13–16].

Handoffs are a complex form of social interaction that occur in “microsystems,” [17] defined as groups of clinicians and staff with a shared clinical purpose and legal responsibility to provide care. Formal “onboarding” training-- the action or process of integrating a new employee into an organization for new physicians in training, is accompanied by informally acquired knowledge and expectations about the day-to-day operation of the microsystem. Differences in local culture have been identified as a major contributor to variations in individual and the microsystem performance and may, in part, explain why interventions that call for rigid adherence to uniform standards have been unsuccessful [18].

Recent literature suggests a change in the conceptualization of handoffs from a mechanical transfer of accurate and complete patient information, to a highly nuanced, contextually sensitive and emergent social-technical communication event [18, 19]. To date, developers of handoff improvement strategies have paid little attention to the complexities of workflow. Competing cognitive, linguistic, technical and physical demands influence the success of a handoff, but are not routinely considered part of the educational process for leaning and improvement [20, 21]. Identifying and enhancing the capacity of clinicians to make sense (clinical sensemaking) of these complexities in patient care is crucial [22]. A sense-making approach that focuses on the motivation of clinicians who are jointly responsible for patient care, rather than focusing solely on errors or root causes, may contribute to the development and implementation of effective and sustainable interventions [23].

The objective of this study was to gain insights into residents’ perspectives on the local cultural assumptions and contextual factors that shape their knowledge about, and skills of enacting effective handoffs. In this paper, we report our findings related to the formal, and importantly, the “hidden” or informal curriculum that residents reported being exposed to in developing these skills.

## Methods

### Setting and participants

We conducted a prospective, multi-method, qualitative study. Data collection included semi-structured cognitive task interviews with medicine and surgery residents at three geographically dispersed VA Medical Centers (VAMCs). Site investigators and chief residents supplied names of potential residents to recruit for a cognitive task interview and to have their handoffs videotaped. Recruited participants were then asked to provide additional names of peers who might be open to participating, a form of snowball sampling [24]. Research assistants met with interested providers, informed them about the study, and obtained their written consent to participate. Residents received a $100.00 gift card for participation in the interview. Ethics approval was obtained from the Indiana University Institutional Review Board and VAMC Research and Development Human Subjects board.

### Data collection

Data were collected from individual Cognitive Task Analysis (CTA) interviews. This method extends the critical decision method of Klein, Calderwood, & Macgregor [25], later adapted by Militello and Hutton, and generally includes a combination of observations and semi-structured interviews [26]. CTA enables elicitation of factors that experienced professional face while engaging in complex clinical tasks [27–30]. Specifically, participants are asked to recall their last handoff and describe 4 to 5 major steps in the process. Cognitive probes were used to explore training/experience, cues, goals, strategies, and information needs and tools used during each step of the handoff process. An interview guide is included as Additional file 1.

The CTA interviews were conducted in a quiet location in the hospital, audio-recorded and transcribed verbatim according to a standardized format. Team members verified that the recordings matched the transcripts an d corrected any transcription inaccuracies.

### Data analysis

Interview data were analyzed using a grounded theory approach to explore key social processes and structures [31]. The interviews were coded using qualitative software NVivo [32]. Emerging codes were circulated among authors and the list of codes was developed into a code-book during face-to-face meetings, conference calls, and email correspondence. Regular conference calls were held to refine the codebook as other codes emerged during the analysis process. In building the inventory of codes, portions of the text and provisional codes were compared and differences reconciled in a series of iterative consensus-building meetings until thematic saturation was reached (e.g., no new codes emerged) [33].

One member of the data analysis team was responsible for updating and maintaining a master file of the consensus coded transcripts (NR). Iterative reviews using the constant comparative method, resulted in assignment of codes to mutually exclusive categories. We report here on the findings related to “training and experience” that were derived from the interviews; study findings on how residents prepare for handoffs have been published elsewhere [34]. A confirmability audit to ensure dependability of the training and experience category analysis was conducted to match code definitions with transcript case examples.

## Results

### Participant demographics

A total of 35 residents were interviewed. Demographics are summarized in Table 1. Data analysis resulted in 2 main themes that emerged: (i) formal and (ii) informal training experiences and three main skills as described below.

**Table 1:** Interviews analyzed by care providers breakdown by study site

	PGY1		PGY2		PGY3		Total
	Medical	Surgical	Medical	Surgical	Medical	Surgical	
Site 1	0	0	7	1	1	0	9
Site 2	12	2	1	2	0	1	18
Site 3	8	0	0	0	0	0	8
Total	20	2	8	3	1	1	35

### Formal training experiences

Formal training strategies included: medical school curriculum (e.g., handoff class, handoff practice, simulation, observation opportunities); resident/ intern orientation handoff boot camp/workshops; and debriefing handoffs to identify necessary information to be included.

### Informal training experiences

Informal, “on-the-job” training experiences were reported as having a stronger influence on actual practices than formal instruction. Residents described three specific handoff-related types of skills: 1) acquiring and applying knowledge to anticipate patient needs and tasks; 2) aligning information needs to work tasks (content and amount); and 3) adapting to the handoff to the setting and context (see Table 2 for a summary of skills and exemplar quotations). We have broken down each of these types into two specific skills.

**Table 2:** Training and experience themes, experiential learning skills and representative quotes related to end-of-shift handoffs

Skill	Sub-skill	Representative quote(s)
Acquire and apply knowledge to anticipate patient needs and tasks	Apply newly acquired clinical knowledge	“When you become an intern and now you’re in a residency, you learn a lot more about what’s important because your knowledge is growing as a doctor.”
	Provide anticipatory guidance and assign tasks	“…Giving them recommendations based on potential scenarios…” “I think now that as I got more experience, I am able to anticipate what problems I see might happen with a patient a little bit better. So, if I foresee that something would go wrong with a patient, I like to tell the cross-cover person you know like ‘Hey, watch out for this guy to go into alcohol withdrawal and if he does, do this.’”
Align information needs to work tasks (content and amount)	Identify pertinent information	“What’s important for someone who’s going to take care of this patient for 14 h needs to know…” “I think it just takes a lot of practice and being on both ends, like receiving and providing to kind of see where the benefits of providing certain pieces of information are, so I think everybody just kind of has to experience it…”
	Be concise	“…Concise is important because there are things. There is a lot. Time doesn’t stand still for handoffs. You’ll be getting pages as you’re getting handoffs…”
Adapting handoffs to the setting and context	Incorporate helpful delivery strategies	“…I really don’t stand during a sign out. If I’m going to give a sign out to a person, I like to sit down and have then sit next to me so we make sure they don’t feel that I’m rushing them, and then give a thorough sign out without…”
	Appreciate others’ styles/preferences for handoff	“…I mean you kind of can read people and when like they’re shutting down. When they’re not listening and they’re writing something else or looking at their phone or like those are kinds of ways.”

#### Skill 1: Acquire and apply knowledge to anticipate patient needs and tasks

Residents recognized that acquiring specific and targeted clinical knowledge continued during residency and influenced their ability to communicate anticipatory guidance and tasks for the upcoming shift during handoffs.

##### Skill 1a. Apply newly acquired clinical knowledge

Residents reported that the quality of their handoffs improved over time as their clinical understanding of patient disease trajectories and medical treatments expanded. Continued learning resulted from month-long rotations on different medical wards and exposure to a variety of cases. Learning also occurred informally when senior residents “intervened” (i.e. ask a question, interrupt to clarify, etc.) during challenging handoffs. One resident (P101) described this type of learning as follows: “*When you first start residency like your senior resident is always there with you when you’re signing out to kind of jump in when the night person asks you a question…”* Additionally, residents developed a better understanding of the implicit language of handoffs: *“I think there is a lot of hidden language in sign out, the way you describe a patient to somebody else,[and] in the way that words are constructed and the sign out itself is constructed.”* (P123).

##### Skill 1b. Provide anticipatory guidance and assign tasks

Anticipatory guidance was reported to provide incoming residents with an early guide to issues that might arise during the next shift. Typically, this type of information included relaying past patient responses to medication or procedures, and communicating clinical trajectories, potential or desired outcomes and care needs*.* For example, anticipatory guidance might include how a patient responded to a specific diuretic dose, how his vital signs varied, reactions and follow-up plans, patient triaging, anticipating nursing questions, tasks to complete, using baseline status for comparison, and alternate care plans if certain conditions occurred during the night shift.

Residents also adopted a simple binary vocabulary for indicating the acuity of a patient’s condition, “sick” or “not sick.” “Sick” referred to patients who could decompensate during the upcoming shift, while “not sick” referred to patients who only needed routine monitoring. The ability to offer anticipatory guidance improved with continued clinical experience and explicit feedback on previous handoffs.

#### Skill 2: Align information needs to work tasks

Residents consistently noted that including the ‘right’ information in the ‘right’ amount was a key and new skill developed to give organized, clear handoffs. One resident (P103) explained that: “*It takes a lot of practice—after you’ve done these sign-outs for months on end, you know the important points to hit on.”*

##### Skill 2a. Identify pertinent information

Residents described the skill of providing patient information as one that best informs the receiver of what they will need to do during their upcoming shift*.* Communicating pertinent information was contrasted with mechanically reciting a problem list. For example, residents discussed how to convey patient acuity by interpreting aspects of seemingly routine updates, such as mental status exam: “*I will ask them those same [mental health] questions overnight, and if things are ‘off’ at all, like that’s gonna tip me off to do more, or if it’s exactly the same, I’ll worry about it less*”. (P118).

One striking example of on-the-job learning emerged as residents completed night shift rotations. Prior to staffing overnight shifts, classroom learning had suggested handoffs were standardized, whether from day to night or night to day. Yet, during “night float” rotations, which refer to when residents cover the night shift, they typically monitored and intervened with patients only when necessary. One resident (P129) described the experiential learning process: “*When you experience the night float … you start knowing what is important, what is not important. So when you put yourself in the call team’s shoes and then as night float, and you know what’s important and what’s not important.”*

##### Skill 2b. Be concise

Residents valued concision and described streamlining the amount of information they included in their handoffs. In this way, they could focus only on items of critical importance. Economy of actions was achieved by limiting detailed descriptions of contingency plans, the amount of background information, and the problem list to 1–2 of the most pressing problems. Compared to start of their internship year, by the end residents reported they had mastered the skill of giving concise handoffs. For example, one resident (P117) stated, *“July 1, it’s like every detail ever and like no consolidation or paring down of anything. It’s just like verbal diarrhea, and you’re like, ‘I don’t know what to make of this,’ I feel like Sherlock Holmes.”* July 1 represents the beginning of residency training in North America. More concise handoffs translated into more effective care by making information actionable during the upcoming shift.

Rather than following a list of prescribed elements learned in didactic sessions, practice had taught them to focus on, and prioritize, the most important and time sensitive information: *“They teach us [that] you need to give a whole hospital course, a whole history of present illness leading up to this hospitalization, …then you go, ‘I’m gonna make sure that I cover all of my bases by talking about everything.’ When you do that, sign-outs take an hour long…”* (P111).

#### Skill 3: Adapting handoffs to the setting and context

Another critical skill related to concision by eliminating redundancy without omitting important information was making adaptations in delivery strategies and accommodating to incoming resident’s preferences and background.

##### Skill 3a. Incorporate helpful delivery strategies

Residents described several different strategies they incorporated into their handoffs including acclimating to noisy environments and providing thorough paper-based back up information. One resident (P125) emphasized the importance of *painting a picture* or relaying *a weird* story to help the incoming resident remember individual patients. Some strategies were taught by senior residents during handoffs; others learned by observing how colleagues handled the process. Helpful handoff strategies varied considerably and were not formally taught. Tips, tricks and shortcuts described by residents did not have the force of institutional authority, or formal rules, regulations and procedures, but rather, they were part of the informal curriculum of resident education.

##### Skill 3b. Appreciate others’ styles/preferences for handoffs

Residents learned from their experience to identify and adjust to other resident preferences for giving and receiving information. For example, residents described variations in the preferred level of detail to be included in a handoff based on previous experiences with other residents. Some also reported adapting their handoffs based on the receiver’s “reputation” or behavioral cues. In both cases, adaptations were based on implicit assumptions about an incoming resident’s preferences: *“Some people are very detail oriented; some people are big picture oriented; some people like to talk and are social during their sign-outs; some people are not.”* (P111) In terms of behavioral cues, some incoming residents used nonverbal cues to indicate that a handoff was too long by diverting attention elsewhere and not listening.

## Discussion

Our findings indicate that critical skills for enacting effective patient handoffs were mostly learned informally through observation and experience. Study participants described how immersion in the local microsystem culture was the best teacher in terms of mastering skills.

Our analysis uncovered six skills viewed by residents as critical to handoff quality: identifying pertinent information, providing anticipatory guidance, applying acquired clinical knowledge, being concise, incorporating delivery strategies, and appreciating styles/preferences for handoffs. Our findings also highlight the dynamic cognitive work required to enact safe and effective handoffs and the importance of shared goals, shared knowledge, and mutual respect between providers. These “relational dynamics” can in turn predict higher levels of quality [35]. These skills require a high level of interpersonal knowledge and sense-making that is critical for accurate assessment of patient status and care needs, care planning, interpersonal and setting-specific issues, and handoff content [22, 23]. Sense-making is the integration of both tacit and explicit knowledge of a situation and is a basis for social action [23]. Our findings demonstrate how residents use sense-making activities to coordinate complex actions like the transfer of rights, duties and responsibilities between providers involved in end of shift handoffs. Too great a focus on content alone can miss the critical role of sense-making and interpretation that “house” the handoff process. Attempts to design effective handoff interventions should balance the urge to standardize with explicating the role of self and situation awareness in handoff interactions [36].

Interestingly, residents did not focus on handoff mechanics, content, or preferred formats. Instead, practical concerns relating to informal organizational culture and experience and how it influenced their workflow made up the bulk of their comments. Our findings suggest that the ability to detect nuanced messages and adapt accordingly is a subtle interpersonal skill that emerges only when a resident has mastered the mechanics of handing off complex patients. Perhaps, only then does the resident have the cognitive resources to attend to the recipient’s skills, attention level, and real-time reactions to the information being conveyed. Residents also reported being less willing to confront each other about handoff defects and inefficiencies which may explain resident self-reported high ratings of their handoffs [4]. Skill acquisition is difficult in the absence of feedback [37, 38]; a goal for future improvement interventions. Safety of patients may also be compromised by a false sense of confidence that emerges when handoff strategies are not rigorously assessed [35].

We have several suggestions for improving handovers based on experiential learning pedagogy. First, changing attitudes may be enhanced through establishing collaborative learning meetings between medical students, interns and residents to better understand each other’s perspectives, competencies (knowledge, skills, and inferences) and expectations [39]. Simulated handoff cases in which ambiguous or incomplete information is communicated could be used to stimulate discussion about sense-making and the tacit (unspoken) rules of social interaction. Direct feedback by incoming resident or by independent monitor during handoff may be greatly beneficial especially during early months of internship or junior residency.

Second, we suggest providing point-of-care education programs that connect best practices with patient safety and efficiency goals [40]. In this way, the hospital providers may better understand how their own thinking and actions impact quality and safety [41]. One approach, used successfully in infection control, is video-reflexive ethnography in which clinicians view video footage of their actions to identify vulnerabilities for spreading infection. This approach allows participants to understand the consequences of their actual behaviors (including those that seem irrelevant), question their habits of practice, and expand reassessment to behaviors outside the video footage (e.g., reduced infections [42] improved handoff communication [43]). Incorporating a video-reflexive session for resident handoffs might reveal non-verbal behaviors, speech pace, and other habits that seemingly are unrelated to effective handoffs.

Third, both classroom and on-the-job mentoring could be enriched by the use of stories or “vignettes” that are meaningful and memorable to residents, because they demonstrate the direct link between handoff processes and their effects on patient care [44]. Incorporating stories and connections is one scaffolding technique for making handoff curricula salient for learners because they demonstrate the direct link between handoff processes and their effects on patient care [41]. In addition, as shown in this study, enacting actual handoffs was viewed as the most effective learning method for developing mastery. Medical students and trainees need “live” opportunities to learn how to conduct handoffs safely and receive feedback.

The study has several limitations. First, data were analyzed and compared at three VA sites with distinct local cultures. Nevertheless, the emergence of the same skills across sites increases the credibility and trustworthiness of the findings. We made all efforts to ensure methodological rigor and validity across the study sites by using a standardized codebook, meeting and talking frequently, sharing and comparing our results. Second the data reflect participant “reports” about their experiences – and could be subject to response bias [45]. Cognitive task analysis is a non-judgmental process that, in principle, reduces, but does not eliminate unwanted bias. Third, the modest number of participants may not be representative of all medical residents’ handoff education or practice.

## Conclusions

Our findings underscore the complex social structure of end-of-shift handoffs with implications for handoff training curricula and efforts to improve effectiveness [46]. The use of individual interviews provided invaluable insights into the subtleties of decision-making, and the underlying shared values, beliefs, assumptions, and norms of that characterize the handoff process. While complex and difficult to define, focusing on sense making and contextual features of handoffs can potentially help residents develop the expertise needed to ensure and safety across care transitions [47].

## Additional file

Additional file 1: Interview Guide. This appendix outlines the interview guide used in conducting CTA interviews. (DOCX 21 kb)

## Abbreviations

ACGME: The Accreditation Council for Graduate Medical Education (ACGME)
CTA: Cognitive Task Analysis
VA: Veterans Affairs
VAMCs: VA Medical Centers
