# Supplementary material for: Content counts, but context makes the difference in developing expertise: a qualitative study of how residents learn end of shift handoffs
Source: BMC Med Educ. 2018 Nov 3;18:249. doi: 10.1186/s12909-018-1350-8 (PMC6215683; doi:10.1186/s12909-018-1350-8)
Supplement: Supplementary file 1 — Interview Guide. This appendix outlines the interview guide used in conducting CTA interviews. (DOCX 21 kb) [file 12909_2018_1350_MOESM1_ESM.docx]

**Additional file 1. Cognitive Task Analysis (CTA) Interview Sample Questions**

**Think about what you did the last time you transferred care for a patient to the next resident at the end of a shift.**

- Please describe what happened by breaking it down into 4-5 major steps*.*
- What is the first major step you recall in the transfer; what is the next major thing you recall in the transfer, etc.
- Starting with the first major step you recall, what were you thinking right at that moment? What cues were you noticing or were influencing your thinking?
- Will you describe the person receiving the handoff and the location of the handoff?
- What goals did you have at that time?
- Did you have everything you needed or wanted to complete the transfer?
- Is there anything in addition you wished you had at that point?
- What did you do to prepare for the transfer?
- Did you feel fully prepared for the transfer? If not, what would have helped?

**The interviewer repeats this type of probing for each of the major steps identified by the resident. After all steps have been probed, additional questions will be asked of the resident if not already elicited by the above interview. For example:**

- Was this a typical transfer of care situation for you? If not, what made this transfer atypical?
- What would have made the transfer better?
- Was the environment in which this handoff occurred typical? How so?
- How did the receiving physician respond to this handoff? Did he/she request more information?
- Do you tailor the handoff based on who is receiving?
  - Ask for examples (i.e., day versus night; within department versus without department; a new intern versus someone you know well; someone with a good reputation, etc.)
- What are the most helpful pieces of information for you to receive when care is transferred to you? (if not already elicited in task diagram portion of interview)
- What questions did you ask during this handoff (ask for roles as both outgoing and incoming)?
- When transferring care for this patient, what information would you share with the incoming resident?
- What information, if any, would you *emphasize* as key points for the incoming resident?
- Does the handoff differ depending on whether you are handing off to day shift or to night shift? Tell me more…
- What errors, if any, would an inexperienced person be likely to make while they are transferring care to the next resident? (E.g. what important information might they overlook, or what less critical information might they overemphasize?)
- Once the transfer is complete what actions (as an incoming or outgoing resident) do you normally engage in (e.g., cognitive review and reflection, re-check status, visit sickest patients)?

**Atypical handoffs:**

- Have you experienced an atypical handoff?
- Do you know when a handoff will be atypical?
- What are the circumstances that led to a handoff becoming atypical?
- How often would you say atypical handoffs occur? How often do they lead to potential errors?

**Training and feedback**

- What kind of feedback have you received about your handoffs?
- Have you ever given another resident feedback about a handoff?
- What does a good handoff look like to you?
- How has your view on handoffs changed as you gained more experience?
- How have your handoffs changed as you gained more experience?

What is your primary goal when you are giving handoff?

What is your primary goal when you are receiving handoff?

What value do you place on carrying out handoffs in comparison to your other work? What do you see as the main objective in performing handoffs?

Thinking back through your education experience, where would you place learning handoffs (in terms of importance?)?

What worries you most about your current handoff practices?

What are 4 – 5 steps you do in order to prepare to RECEIVE handoffs?

What do you see as key areas of improvement in terms of handoffs? What advice would you give to other residents who are seeking to improve how they give and receive handoffs?

**Now think about what you did the last time you received a transfer of care from another resident at the end of a shift.**

The interviewer will repeat same sequence from above for the major steps that are identified.
